# On Medico-Legal Evidence in Cases of Insanity

**Published:** 1855-01-01

**Authors:** Forbes Winslow


### LETTSOMIAN LECTURES.

### No. IV.

### ON MEDICO-LEGAL EVIDENCE IN CASES OF INSANITY.

(CONCLUSION.)

*Delivered before the Medical Society of London.*

By FORBES WINSLOW, M.D., D.C.L.

I PURPOSELY decline entering at any length into the consideration of the law of lunacy relating to dispositions of property, and the performance of the marriage contract. In the former case the proof of insanity invalidates all testamentary documents; but the courts are extremely jealous in interfering with the apparent wishes of the testator, unless clear and positive lunacy be established. The character of the testamentary document itself is generally viewed as the most important evidence of the capacity of the attesting party. Persons actually in confinement, and so violent as occasionally to require the application of mechanical restraint, have executed wills, and such wills have been declared valid and operative in the Prerogative Court; the principle of law being, that the *testament itself* exhibited, *primâ facie*, no evidence of mental derangement. If the will should be such a will that a sane and rational man would make—the property descending in the right and legitimate channel—the court will not easily be induced to set it aside, even if a considerable amount of eccentricity, oddity, and even insanity, have existed. The proof of eccentricity to an extreme degree, even if accompanied by a testamentary disposition contrary to the usual order of succession, is not sufficient to induce the Ecclesiastical Court to pronounce a will invalid. The following remarkable case of Morgan *v.* Boys is one in point:—

The testator in this instance died, leaving by his will a large fortune to his housekeeper. The will was disputed by his relatives on the ground that it bore intrinsic evidence of his not having been in a sane state of mind. After having bequeathed his property, the deceased directed that his executors should cause some part of his bowels to be converted into fiddle-strings, that others should be sublimed into smelling salts, and that the remainder of his body should be vitrified into lenses for optical purposes! He further added in a letter, "the world may think this done in a spirit of singularity or a whim;" but he expressed himself as having a moral aversion to funeral pomp, and he wished his body to be converted to purposes useful to mankind. Sir Herbert Jenner Fust, in giving judgment, held that insanity was not proved; the fact merely amounted to eccentricity, and on this ground he pronounced in favour of the will. It was proved that the testator had conducted his affairs with great shrewdness and ability; that he not only did not labour under imbecility of mind, but that he was treated as a person of indisputable capacity by those with whom he had to deal.

The medical man has occasionally to give evidence as to the existence of what, in legal phraseology, is termed a "*lucid interval.*" Without entering into a psychological or pathological consideration of this *vexata quæstio*, I will only observe, that all who have had opportunities of studying insanity, must readily admit, that during attacks of mental derangement, the mind does occasionally become exempt from the influence of diseased impressions—at least, from all obvious and appreciable delusions, and is quite competent at these periods of intermission, to the exercise of a right judgment in relation to the disposal of property.

With regard to the legal bearing of this subject upon the question of marriage, it must be obvious that insanity must invalidate the most important contract of life, the very essence of which is consent. The spiritual court has the sole and exclusive cognizance of questioning and deciding directly the legality of marriage, and of enforcing specifically the right and obligations respecting persons depending upon it. But the temporal courts have the sole cognizance of examining and deciding upon all temporal rights of property; and so far as such rights are concerned, they have the inherent privilege of determining incidentally either upon the fact or legality of marriage.*

In cases of disputed wills, on the ground of mental incompetency, the evidence of the medical witness is generally recorded (privately) before a proctor or his representative. The witness has to reply to a series of written interrogatories relating to the

* Starkie on Ev.

testator's state of mind, and his replies are written at length by a person specially deputed to examine him, and take his evidence. The cross-examination is also conducted upon the same principle, and the evidence thus recorded, after being attested upon oath, is adduced in court during the trial. In attempts to invalidate the marriage contract upon the ground of insanity, the inquiry is in some cases of the nature of an ordinary commission of lunacy. Should the insanity be thus established, the subsequent question of divorce must of necessity come before the Ecclesiastical Court. It is not, however, indispensable that in these cases a writ *De Lunatico Inquirendo* should issue.

I now proceed to consider another division of the subject,—viz., that relating to the question of capacity to manage both the person and property, and to submit to you an outline of the character of the evidence adduced during the prosecution of a writ *De Lunatico Inquirendo.* It is at these important inquiries that the legal and medical signification of the terms "soundness" and "unsoundness" of mind come legitimately under consideration. Let me briefly refer to the legal import of these obscure and much-vexed phrases, as defined in one of the recognised text-books upon the "Law of Lunacy."

"A sound mind," says Shelford, "is one wholly free from delusion, all the intellectual faculties existing in a certain degree of vigour and harmony, the propensities, affections, and passions being under the subordination of the judgment and the will, the former being the controlling power, with a just perception of the natural connexion or repugnancy of ideas. Weak minds, again, differ from strong in the extent and power of their faculties; but unless they betray symptoms of a total loss of understanding, and of idiocy, or of delusions, they cannot be considered unsound.

"An unsound mind, on the contrary, *is marked by delusions,* mingles ideas of imagination with those of reality, those of reflection with those of sensation, and mistakes the one for the other; and such delusion is often accompanied with an apparent insensibility to, or perversion of, those feelings which are peculiarly characteristic of our nature. Some lunatics, for instance, are callous to a just sense of affection, decency, or honour; they hate those without cause who were formerly most dear to them; others take delight in cruelty; many are more or less affected at not receiving that attention to which their delusions persuade them they are entitled. Retention of memory, display of talents enjoyment in amusing games, and an appearance of rationality on various subjects, are not inconsistent with unsoundness of mind; hence sometimes arises the difficulty of distinguishing between sanity and insanity. The man of insane mind from disease, having been once *compos mentis,* pertinaciously adheres to some *delusive idea,* in opposition to the plainest evidence of its falsity, and endeavours by the most ingenious arguments, however fallacious they may be, to support his opinions."*

Previously to the time of Lord Eldon, the term unsoundness of mind, and its equivalent, "unsound memory," were used indiscriminately in several of the old statutes, not only synonymously with the word lunatic, which in its strict legal signification means a disease of the mind with lucid intervals, but with the phrase idiocy, or permanent insanity. It was reserved for Lord Eldon to give importance and significance to this phrase. Lord Chancellor Hardwicke maintained, that the term implied, not mere weakness of understanding, but a total deprivation of sense Lord Eldon says: "Of late, the question has not been, whether the party be insane, but the court has thought itself authorized to issue a commission *De Lunatico Inquirendo,* provided it is made out, that the party is unable to act with any proper and provident management—liable to be robbed by any one—under imbecility of mind not strictly insanity, but, as to the mischief, calling for as much protection as actual insanity." Again, his lordship observes, "that unsoundness in some such state of mind undistinguished from idiocy and from lunacy, and yet such as makes him a proper subject for a commission." The legal acceptation of the term unsoundness is, as Mr. Amos observes, "not very easy to define, for it is neither lunacy, idiocy, imbecility, or incompetency to manage a person's own affairs; and yet, we have seen, an inquisition finding a person unfit to manage his own affairs, and therefore not of sound mind, has been found bad." Mr. Amos concludes his remarks by stating that "unsoundness of mind is a legal term, the definition of which has varied, and cannot, even in the present day, be stated with anything like scientific precision." Mr. Shelford regrets that Lord Eldon should have departed from the original signification of

* Law of Lunacy, by Leonard Shelford, Esq. 1847.

the term unsoundness of mind, and that so much uncertainty and latitude should have been given to the phrase. In a subsequent case, Lord Eldon appears to have laid down a clearer view of his meaning in relation to this important matter. He says: "Whatever may be the degree of weakness or imbecility of the party to manage his affairs, if the finding of the jury is only that he was of an extreme imbecility of mind, that he has an imbecility to manage his own affairs, if they will not proceed to infer from *that*, in thus finding upon oath, that he is of unsound mind, they have not established, by the result of their inquiry, a case in which the chancellor can make a grant, constituting a committee, either of the person or estate. All the cases decide that mere imbecility will not do, unless that imbecility, and that incapacity to manage his affairs, amount to evidence that he is of unsound mind, and he must be found to be so." The *dicta* of Lord Chancellor Eldon have, however, been often disregarded by his eminent judicial successors; and in a statute of William IV., relative to trustees and mortgages, a power is given to the Lord Chancellor to issue a commission "*De Lunatico Inquirendo*" in all cases in which *an inability to manage affairs can be established, apart altogether from the existence of idiocy, lunacy or insanity.* So much for the glorious uncertainty of the law, and the civil security of the subject!* It would appear that the term "unsoundness of mind," although a recognised and adopted phrase, is to be considered as a legal phantom—an *ignis fatuus*—a condition of mind amenable to no philosophical or judicial analysis, incapable of being submitted to any psychological test—like a Will-o'-the-wisp, for ever eluding our grasp, and placing at defiance every standard of comparison—a phase of diseased understanding—a form of lunacy suspended upon, or hovering

* Dr. Ray, when referring to the facility with which commissions of lunacy are granted in this country, remarks: "One finds it difficult to believe on what slight grounds interdiction is there (in Great Britain) every day procured—a measure that, with the ostensible purpose of protecting the interests of the insane party, is too often, in reality, designed to promote the selfish views of relatives and friends. A kind and degree of mental impairment that has never obscured the patient's knowledge of his relative situation, never altered his disposition to be kind and useful to those around him, never weakened his enjoyment of social pleasures, and never affected his capacity to manage his concerns with his usual prudence, has been repeatedly deemed a sufficient reason for depriving him of the use and enjoyment of his own property, and subjecting him to all the disabilities which the law can impose."

between, the confines of positive mental alienation and complete idiocy—an intermediate state of existence—a kind of *tertium quid*, to which modern jurists appear incapable of attaching any precise, definite, or philosophical meaning. Nevertheless, in our courts of law it is *no* fiction—it assumes a palpable form—it is an accepted term—an admitted phrase; and, as medical witnesses, we must be prepared to be asked the question, whether we are of opinion that the case in reference to which we are examined is one of unsoundness of mind, and whether that unsoundness of mind is or is not associated with an incapacity to manage the person and property? It is our duty, however, to recognise no form of mental unsoundness which is not *positively the product of disease*. The judges of the land may affix their own interpretation to the phrase, "unsoundness of mind;" but, as medical psychologists, we must never forget what is due to our position as witnesses, as well as what we owe to the profession, and to the cause of *truth*, and resolutely repudiate any other definition of the term than that justified by a strict psychological analysis.

Unsoundness of mind is either a "diseased" or "healthy" condition of the intellect. If the term implies only *natural mental decay* (unassociated with any well-marked symptoms of disease of the brain, the excitement of insanity, or delusive impressions), a condition of mind occasionally exhibited by persons of healthy intellect, the mental infirmity often contemporanous with old age; if it refers to an incapacity and inaptitude for the performance of the ordinary business affairs of life, and which may exist apart altogether from connate idiocy or acquired imbecility, insanity, or lunacy; then WE have no authority to take cognizance of the condition—it does not legitimately come within *our medical* jurisdiction. If we accept the phrase "unsoundness of mind," we can attach, *medically*, no other signification to it than that of a *mind in an unhealthy condition*. Admitting this to be a rational view of the matter, it will be our duty to consider the term as synonymous with insanity, aberration of mind, or lunacy. We cannot admit the existence of a *legal*, apart from a *psychological*, unsoundness.

In making this distinction, I do not wish to prejudge the important question, as to whether there are not states of the intellect clearly accompanied by an incapacity to manage both the person and property, the result of a premature and natural decay of the mental faculties, independently of any physical alteration in the nervous matter which would justify us in bringing the person so affected within the wise and protective influence of the law? It is quite possible that in some conditions of the mind, "*interdiction*" and "protection" may be desirable for the purpose of guarding the person and property of the individual, who could not, without an act of great injustice, and a monstrous and cruel perversion of the law and of science, be pronounced to be, in the right acceptation of the term, either insane, imbecile, or a lunatic. Should such a class of cases be recognised by statute, and made the subject of legal inquiry and protection, it will be necessary for us to adopt proceedings very dissimilar to an ordinary commission *De Lunatico Inquirendo;* neither should we be justified in applying to those so brought within the jurisdiction and control of the law, the terms usually adopted in writs of this description: such as lunatic, imbecile, idiot, or unsoundness of mind.

There are upon record cases of this kind, which have been made the subject of judicial inquiry. In the case of Ridgway *v.* Darwin, a commission of lunacy was supported against a person who, when sober, was a very sensible man, but being in a constant state of intoxication, he was pronounced incapable of managing his property. This liberality of courts of justice is clearly at variance with the *dicta* of Lord Coke, who pronounced the drunkard to be "*a voluntarius dæmon.*" By the Roman law, if a man by notorious prodigality was in danger of wasting his estate, he was considered as *non compos,* and committed to the care of *curators* or tutors, by the prætor. By the laws of Solon such prodigals were branded with perpetual infamy. Blackstone questions the propriety of the Roman and Grecian law with regard to drunkards and spendthrifts. He says, it was doubtless an excellent method of benefiting the families, but it hardly seems calculated for the genius of a free nation, who claim and exercise the liberty of using their own property as they please. "*Sic utere tuo ut alienum non lædas,*" is the only restriction our laws have given with regard to economical prudence.

The medical witness deputed to ascertain the state of mind of a party, prior to the presentation of a petition to the Court of Chancery for the issuing of a commission *De Lunatico In-*

*uirendo,* is required to prepare for the consideration of the Lord Chancellor, an affidavit embodying the facts, and his opinion of the case. I would advise the witness not to remain satisfied with one examination of the alleged lunatic, even if the insanity should be very apparent and obvious. The court is better satisfied if the affidavit of the medical *expert* is based upon several interviews. The opinion of the witness assumes a legal form whilst in the hands of the solicitor, and the party giving it, is required to appear at the affidavit office, or before one of the commissioners appointed by the Lord Chancellor, to administer oaths in Chancery, to swear to the accuracy of the document. It is very important that the medical witness should, at the moment of the examination or immediately afterwards take *full notes* and accurate dates of every conversation with the person whose state of mind is likely to be the subject of investigation. If called upon to give *vivâ voce* evidence, he will be permitted to refer to these memoranda, if made at the time of examination. It should also be borne in mind, that the opposing counsel and judge (if the commission be contested) have a right to see and examine, in open court, the notes of the medical witness. Before being called upon to give evidence at a commission of inquiry, he is generally expected, by repeated interviews with, and examinations of, the alleged lunatic, to have made himself fully acquainted with all the peculiar and characteristic features of the case, and to have satisfied his mind as to the existence, not only of mental derangement, but of insanity associated with an inability, from disease, of managing both the person and property. In our examination of the alleged lunatic, we must not take for granted every statement alleged against him; but it is our duty to investigate for ourselves into the truth of the representations made for the purpose of establishing a case of insanity against the person whose capacity and sanity of mind we are deputed to examine. In the generality of instances, the delusions of the party are apparent, and we have little or no difficulty in detecting the mental derangement. In many cases, the intellect is reduced to a sad state of imbecility; and in this type of insanity we have no obstacles to interfere with our arriving at a right conclusion; but doubtful instances occasionally are brought under our notice, giving rise to considerable anxiety, and requiring for their scucessful exposition great caution, much time, and patience. Delusions are sometimes cunningly concealed for a length of time and notwithstanding we are certain that they exist, no amount of ingenuity will induce the patient to disclose them, particularly if made aware of the object of our visit. I had recently to see a lady whose insanity was manifested in a remarkable degree in her every action; but after paying her several visits, I found it impossible to induce her to exhibit any one delusive impression or insane idea; but no sooner had I left the room, than her conversation and conduct became outrageously insane. Many insane persons are able to talk with apparent rationality, but cannot write without exhibiting their insanity. I have examined recently one very remarkable case of this kind, in a clever, well-read, and intellectual woman, whom I had occasionally to visit. I never could detect the slightest aberration of mind in her conversation, and yet almost invariably upon my leaving, she placed in my hands a letter (which had been written previously to my calling), full of the most absurd extravagancies and fancies; accusing strangers, myself, and the members of her family, of being engaged in a deeply-concocted conspiracy against her property and life. Several of these peculiar and interesting cases are recorded, and the medical man has been advised, with the view of obtaining an insight into the true condition of the mind, to open a correspondence with the alleged lunatic, upon the principle that few persons positively insane can, for any length of time, write, without exhibiting their delusions, whatever amount of self-control they are able to exercise over their thoughts and morbid ideas, during protracted conversations. It is essential for us to ascertain the degree of knowledge possessed of the ordinary and every-day occurrences of life. Upon one occasion I was conversing with a person whose state of mind was the subject of investigation, and finding him rational, and apparently sane upon all points, I questioned him as to who was the reigning sovereign, without knowing he had any delusion upon the point. The person immediately started from his chair, exclaiming, in an excited tone of voice, "I am the sovereign!"

It is a usual practice to test the alleged lunatic's knowledge of the elements of arithmetic, and to ascertain whether he has any idea of the ordinary rate of interest obtainable for money in the funds, or other modes of investment. It would also be desirable to place before him a simple sum of addition and multiplication. The medical witness may be asked whether he has pursued this mode of examination, particularly in cases of impairment of mind and imbecility occurring early in life. On this account I bring these apparently trivial and unimportant matters before you.

Upon one occasion the mental incapacity of a party was clearly exhibited, by his being easily induced, in the presence of his solicitor, to write the physician who examined him a check for £500, in payment for some imaginary service that had been rendered him. It was palpable that a man who could thus commit himself with a stranger, would be the willing dupe of any designing person who might be disposed to take advantage of his mental infirmity, and therefore was quite unfit for the management of his person or property. The "arithmetical test," as it is termed, is, in cases of doubtful insanity, of no value *per se*. It is only when conjoined with other evidences of mental impairment and admitted incapacity, that any importance should be attached to it. The position in life of the party, the amount of education he has received, his age, and the opportunities which have been afforded him of acquiring information respecting the ordinary commercial or business affairs of life, should invariably be considered whilst testing the capacity.

In commissions of lunacy, the witness must not only be prepared to give an opinion as to the then state of mind of the party, and competency to take care of his person and manage his affairs, but he must be prepared, occasionally, to pronounce judgment as to a prior questionable condition of brain and mind. The alleged lunatic may, under the exercise of undue influence, have previously alienated his property by will, or been induced to execute other important documents. The witness will be called upon to depose as to the probable state of the brain at the time, and as to the length of the alleged existing attack of insanity. Well-marked symptoms of organic cerebral disease may be present; and it will, in some cases, be an important point to decide, whether such a condition of physical ill-health has not been of some years' duration, impairing the mental vigour, destroying all power of rational conduct and healthy continuity of thought, and thus interfering with a right exercise of the judgment and affections, in the legitimate disposal of property.

The witness, in giving evidence, must abstain from the use of pedantic terms, and technical phraseology. The more simple, unaffected, and unadorned his statement, the greater will be its moral weight. He should carefully and scrupulously avoid all *positiveness* and *dogmatism*, and his testimony ought to be accompanied with judicious qualifications, when relating to cases of difficulty, doubt, and obscurity, respecting which there may, even among eminent scientific men, be great discrepancy of opinion. Dr. W. Hunter, when speaking of the confidence placed in the evidence of men of science, observes, "Some of us are a little disposed to grasp at an authority in a public examination, by giving a quick and decided opinion, which should have been guarded with doubt; a character which no man should be ambitious to acquire, who, in his profession, is presumed every day to be deciding nice questions, upon which the life of a patient may depend."* The evidence of the medical *expert* should impress the court with the conviction that his opinion has not been hastily, crudely, indiscreetly, or rashly formed. It should appear as the result of a full, careful, deliberative, and scientific consideration of the case. Having a lucid conception of the nature of the evidence he is prepared to give, the witness should quietly, but manfully and firmly, maintain his position, and not permit himself to be confused or driven from his point by the cunning artifice of counsel, or thrown off his guard by the disingenuous remarks of the judge. A medical witness, whilst under examination respecting the grounds upon which he had signed a medical certificate of lunacy, after having stated very fairly his reasons for so doing, was subjected to a close examination. He replied to the interrogatories to the best of his ability, rigidly adhering to the simple facts of the case. The answers to the questions did not appear to satisfy the counsel, and he exclaimed, in a pet, "*That* (referring to a particular reply) is not the answer *I wish.*" The proper and immediate rejoinder was, "I know not what reply *you wish*, but it is the only one I have the power of giving, and the only one I can give, consistently with my view of the facts of the case." In the celebrated Bainbrigge Will Case, tried at the Stafford Assizes, a physician, whilst under examination, was asked a question respecting monomania.

* On the Uncertainty of the Signs of Murder. By Dr. W. Hunter.

He replied to the interrogatory, coupling with his answer an observation, that he was of opinion that cases of pure monomania did not exist. The judge immediately interposed, and stopped the witness, observing, rather sharply, that he (the physician) was well acquainted with the legal and generally received definition of monomania, and he must adhere to that, for the court could not listen to any metaphysical or psychological discussion about the term. "Monomania," said the judge, "implies a delusion upon one point, the mind being apparently sound and sane upon all others." It would be well for the witness to avoid such altercations, and never permit himself to be involved in a metaphysical disputation. No good can result to our own character, or to the party in favour of whom we appear, by thus entangling ourselves in a philological dispute with the judge, or by attempting any precise medical or psychological definition of terms. Whilst strongly recommending the witness to maintain a firm and manly bearing, I would at the same time caution him against the attempts, if such should be made, to involve him in personal altercations with counsel. It will often be his duty, when under examination, to exercise great self-command, amidst extreme irritation. He should never lose his temper, or indulge in witticisms or retorts upon counsel, even if a happy occasion should present itself for a display of such repartees or pleasantries. An apothecary, who had previously acted as clerk to a barrister, was, whilst under examination in one of the courts in Westminster Hall, asked to inform the court, how long he had changed his position in life? The witness replied, "I began the study of medicine at a much earlier period of life than the late Lord Erskine did that of law, and he attained to far greater eminence in his profession than ever you will!" The judge did not forget this piece of impertinence; for, when alluding to the evidence of the apothecary, he observed, "that whatever knowledge that witness had obtained in studying his *two* professions, it must be clear to every one, that he had not acquired a knowledge of *manners.*" These injudicious attempts to "*trim* the lawyer," to "*set* him down," and to "fight him with his own weapons," almost always recoil upon the witness. A carpenter was under examination in reference to a serious affray of which he had been cognisant. He was asked, how far he was from the spot at the time of the occurrence? The witness stated the distance with minute exactness, even to the fractional part of an inch. Being then asked, what induced him to qualify himself to give so singularly minute and precise an answer, he replied, "that, thinking some fool might ask him the question, he had taken the precaution of accurately measuring the ground." This was viewed at the time as a happy *hit;* but it would seriously damage the weight of scientific evidence, and interfere with the legitimate course of justice, if witnesses were allowed, even under admitted provocation, to thus unseemly conduct themselves whilst assisting in the solemn administration of the law.*

Should counsel be disposed, not for the purpose of eliciting the truth, but with the evident object of puzzling and confusing the witness, unconsciously impaling him upon the horns of a metaphysical dilemma, designedly subject him to an unfair examination upon abstract points, thus purposely placing him in a ridiculous position, and damaging his testimony, I would advise the witness respectfully to refuse to reply to the questions, intimating to the court that he was of opinion that they had no direct reference to the point at issue, and could not, in his opinion, throw any light upon the nature of the case under consideration. I will, with the view of conveying an idea of the kind of metaphysical disputation to which a medical witness has occasionally to submit, cite a portion of the examination of a psychological *expert* in a case of disputed insanity.

Q. What would you call insanity? A. Some derangement of the intellectual faculties, or of the passions, either general or partial.—Q. What do you call a derangement? A. An alteration from a natural or healthy state.—Q. What do you call the intellectual faculties? A. The faculties by which we reason, compare, and judge.—Q. What do you call the affections and

* Dr. Bankhead, the private physician to the late Lord Castlereagh, when giving evidence in a case of great importance, was subjected by the counsel, then Mr. Brougham, to a severe cross-examination. The Doctor, in reply to a question, gave an answer which was not deemed at all satisfactory. Mr. Brougham, looking steadfastly at the witness, held up his finger, and pointing it significantly at him, repeated in a measured tone of voice the interrogatory. Dr. Bankhead appeared much irritated at Mr. Brougham's mode of elevating his finger, and manner of repeating the question, and he immediately clenched his fist and shook it at the counsel. Mr. Brougham requested that the witness should inform the court why he assumed so menacing an attitude. He replied, that "it was his practice, whenever a gentleman pointed his finger at him, to shake his fist in return."

passions? A. They are called the motive powers or faculties.—Q. What are the intellectual faculties? A. Comparison, judgment, reflection.—Q. What is comparison? A. By comparison we compare two or more things with each other.—Q. What is judgment? A. Judgment enables us to choose between two or more things after comparison has done its work.—Q. What is reflection? A. The comparison and judgment bestowed upon a subject.—Q. Where do you find the faculty of judgment described? A. I have not given it from any author whom I can name.—Q. Is there any such faculty as the will? A. I don't know that the will could hardly be called a faculty.—Q. What is it? A. The will is a power—a determination of the mind to do something. I wish to avoid going into a metaphysical discussion.—Q. What kind of a power is the will—physical or mental? A. It belongs to the mental powers.—Q. What is the difference between the mental powers and the intellectual faculties? A. I don't make any difference.—Q. Then do you call the will an intellectual faculty? A. It does belong to the faculties of the mind. I do not think it is very properly called a faculty: a good many things go to make up the will.—Q. Where does it operate from? A. I should be glad to avoid any metaphysical discussion about the will. I am not now prepared to go into it. The will is an operation of the mind. If the passions and affections are in action, they determine the individual to do something, and that is called the will.—Q. Is the will passive, then? A. I cannot say that it is passive; I should call it active. The intellect directs the determination to do something, and that determination is the will.—Q. But what part do the passions perform? A. The will is an operation of the mind; the passions and affections determine the act. The will is the result.—Q. What has judgment to do with the will? A. It directs the will. It takes both judgment and the will to choose.—Q. What is reason? A. Reason is an exercise of the intellectual faculties.—Q. Is reason a faculty of the mind? A. I should not call it a faculty; it embraces several faculties—memory, comparison, judgment, and some others, all form the reason.—Q. Have you any experience in the treatment of the insane? A. I have not. I have seen many in the almshouses at Philadelphia.—Q. Have you seen persons that you would not know to be insane from observation? A. Yes; and I have seen those that I should not know to be insane without being told.*

Many witnesses seriously commit themselves by an undue *loquacity*. This fault — and it is a prevalent and a very serious one—cannot be too rigidly guarded against. Keep to the text; answer the questions tersely, and epigrammatically; and if you should be called upon for a further explanation, let it be brief, and to the point. "I have heard," says Dr. Gordon Smith, "a very eminent lawyer, after putting a peremptory interrogation to a witness, add, with much energy, 'Now, sir, that is my question, and I will have an answer *yea* or *nay!*' It is not very likely that such an overbearing manner will often be observed towards us; but something allied to it might be shown by an advocate, who, having framed a question especially to suit a particular purpose, might not be inclined to trust the discretion of the witness, or disposed to risk any other answer than that he has baited his question for. Our business must be to inform the *court* and the *jury* of the *truth* of the matter, and to disregard the *tenour* of the question, when it is apparent that it is not intended to elicit the truth, still more so if its obvious bent is to disguise it."

The witness should carefully divest himself of all appearance of partisanship. A quiet, calm, respectful demeanour —and a cautious and modest expression of opinion, even in cases which admit of no doubt — always convey a favourable impression to the court, and give additional weight and influence, to medico-legal evidence. He should remember that in all probability the course of examination is carefully prepared, it being the object of the advocate to obtain from him a reply to a *consecutive series of questions*, thus gradually unfolding and eliciting the truth. Should he, in his eagerness and anxiety to make a favourable impression upon the court, anticipate the interrogatories, he might seriously interfere with the conduct of the case, and injure the cause he is most anxious to uphold.

It occasionally occurs that a medical witness may be fully competent to give sound and satisfactory evidence in relation to the presence of insanity, without having the power of clearly

* The trial of W. Freeman, for the murder of John G. Van Nest, Auburn. 1848.

stating the grounds for his opinion. A medical gentleman, upon being asked, whether he considered a certain person of unsound mind, replied that such was his belief. He was then requested to state his reasons. He said he had formed his conclusion from the "general manner," and "deportment of the patient." The witness was then asked, to describe the "manner," and "deportment," to which he referred. He replied that the patient was "odd in his manner, and had an insane and peculiar appearance about his eye and countenance;" but upon being closely pressed by counsel to describe these symptoms more minutely to the jury, the witness was at once nonplussed, became embarrassed, and broke down. He had a lucid and a *right* opinion of the *matter of fact*, but had no power of describing the symptoms from which he had formed his conclusions. Many men are fully able to give testimony as to *results*, but are totally incompetent to explain the process of reasoning, or succession of thought, by which they have been led to the deduction. A man of practical good sense, who, upon being appointed governor of a colony, had to preside in its court of justice without previous judicial practice or legal education, received the following advice from Lord Mansfield: "Give your decisions boldly, for they will probably be right; *but never venture on assigning reasons, for they will almost invariably be wrong.*" Lord Mansfield knew, says Mr. Mill, who relates the story, that if any reasons were assigned, they would necessarily be an after-thought, the judge being *in fact* guided by impressions from past experience, without the circuitous process of framing general principles from them; and that if he attempted to frame any such, he would assuredly fail.* It would not be difficult to account, psychologically, for a defect of this kind. Are we not daily in the habit of meeting men who have, in relation to matters of art, &c., an *intuitive* perception of the true and beautiful, but who have no power of describing or analysing their sensations and perceptions?

A favourite manœuvre of counsel is to ingeniously construct a number of hypothetical cases, apparently illustrative of the point at issue, and to place them *seriatim* before the witness, with the view of obtaining his opinion of each individual symptom of the alleged mental condition. The replies to such interrogatories, if

* System of Logic, by J. Stuart Mill, vol. i. p. 254.

unguardedly expressed, are often subsequently referred to, for the purpose of damaging his evidence. We should protect ourselves from these legal onslaughts, by carefully considering, before we commit ourselves to an answer, the precise bearing of every interrogatory; it must be rapidly viewed in all its relations, and if we are not thoroughly satisfied as to its character, it is our duty to request the counsel to repeat the question. If we do not clearly perceive its tendency, we must protect ourselves, by carefully qualifying our answer. In a case where the validity of a will was contested, on the ground of the insanity of one of the subscribing witnesses, it appeared in evidence that he had at one time entertained some absurd delusions, and had attempted suicide; but that for a few months prior to the execution of the will he had repudiated the delusions, quietly pursued his studies, had written a book, and in fact was apparently well, with the exception of his being unusually shy, with a desire for solitude. To one of the witnesses, who had spoken in favour of the sanity of the party, the following question was put:—"Supposing he had committed murder about the time he had witnessed the will, would you have considered him as morally responsible for the act?" This question is said to have been artfully founded upon the imputed disposition of the witness to admit too readily the plea of insanity in criminal cases. The court would not allow the question to be answered, but the reply would not have promoted the object of the counsel.*

In giving evidence, it is necessary to remember that the counsel is not permitted to ask the witness to form an opinion of the condition of mind from the testimony of others. As far back as 1760, Lord Hardwicke, then sitting as Lord High Steward at the trial of Earl Ferrers, decided that such evidence was not legally admissible. A witness, he declared, could not be asked whether the facts sworn to by other witnesses preceding him amounted to insanity; he may be asked if such and such symptoms were, in his opinion, indications of insanity, but the witness cannot be removed from the witness into the jury-box. Evidence of this character is admitted in American courts of law. In the case of Hawthorn *v.* King,† the question of the sanity of a tes-

* American Journal of Insanity.
† Massachusetts Reports, vol. viii. p. 371.

tator was tried, and the counsel for the appellant moved that the attending physicians should be allowed to state whether, in *their opinion*, the deceased, at the time of executing his will, was of sound and disposing intellect. This was objected to, on the ground that the *sanity of the party must be determined by his conversations and actions.* These were said to be the only standard. It was alleged that if such a question were put to the physicians, it would be placing them in the position of the jury. The court, however, took a more liberal view of the matter; and considering very properly that the *truth* was the great and ostensible object in view, overruled the legal objection, and allowed the question to be asked, stating that the medical witnesses would be permitted to give their reasons for any opinion they might entertain.

All attempts at a *definition* of insanity should be avoided.

——— "For to define true madness,
What is't? but to be nothing else but mad!" *

The legal profession is too disposed to regard all judicial investigations involving the question of mental capacity, as they do proceedings at *nisi prius;* and under, I have no doubt, a conscientious appreciation of their functions as advocates, often strive their utmost to destroy, if possible, the opposing medical testimony. Knowing the obscurity of the subject, and the difficulties with which the medical witness has to contend, in giving an accurate definition of insanity, the counsel most unfairly endeavours to pin him down to one; and then, by demonstrating its fallacy, overthrow the whole moral effect of his testimony. If asked to define insanity, it will be more judicious at once to candidly acknowledge our utter incapacity to comply with the request, than, by a vain and ostentatious display of metaphysical lore, to peril the life and interest of a fellow-creature.

There are two principal modes of establishing the existence of insanity during investigations under a writ *De Lunatico Inquirendo;* first, by proving the existence of a *specific* delusion; and, secondly, by showing that the party was guilty of a series of acts of extravagance, in opinion and conduct, originating in unsoundness of mind. The first is the most satisfactory and conclusive kind of evidence; and, when clearly

* Shakespeare.

established, carries conviction to the judgment of the court. When the proof depends upon the existence of a series of extravagancies, the witness must protect himself against a common mode of legal procedure. A number of acts of eccentricity and oddity, both in ideas and conduct, are detailed by him, from which he very rightly, and justly, infers the existence of unsoundness of mind. Viewed collectively, these afford irrefragable evidence of a certain questionable mental condition; but in the cross-examination, counsel, by a well-known mode of legal analysis, skilfully separates the whole conduct of the supposed lunatic into detached portions or sectional divisions; and putting each extravagance, eccentricity, and oddity (alleged to be symptomatic of insanity) *seriatim*, to the witness, inquires, whilst specifying such *individual characteristic symptoms*, whether each one, considered independently of the others, is, in his estimation, a proof of incapacity, insanity, or unsoundness of mind; and thus, unless conscious of the designs of the advocate, the witness may be reduced, by his replies, to the necessity of renouncing his previously expressed opinions; or of absurdly maintaining them after all the facts upon which they are based are knocked from under him by the cleverness and ingenuity of counsel!

Refusing to involve himself in a metaphysical disputation, by declining to give a definition of insanity, the witness will, in all probability, be asked, what is insanity, and by what process of reasoning he has arrived at the conclusion that the party respecting whom he is giving evidence is incompetent for the government of himself and his affairs, or is of sound, or unsound mind? In reply to such interrogatories, it is sufficient for him to say, generally, that he has formed his judgment of the condition of mind by the *conduct*, *conversation*, and *ideas* of the person; by considering the symptoms of the case in the aggregate, specifying, of course, the morbid peculiarities of conduct, and the character of the delusive impressions. By this general mode of recording his opinions, the witness will protect himself from a legal snare often laid to entrap and embarrass him.

But whilst suggesting the avoidance of all definitions of insanity, I consider it necessary to recommend the witness to be prepared to answer satisfactorily any questions that may have reference to the scientific import of the terms ordinarily referred to in these judicial inquiries, to designate recognised legal forms of insanity—viz. *delusion*, *idiocy*, *dementia*, and *imbecility*, &c. I have often been amazed at the answers received by counsel to questions of this character, and given, too, by witnesses of known experience and established reputation. A medical gentleman of some position, whilst giving his evidence very recently in a disputed commission of lunacy, in answer to the question of counsel, defined *idiocy* to be "*inertness of mind.*" The acute lawyer made the most of this unfortunate definition; and feeling that he had within his grasp a witness who used terms without having any clear idea of their signification, tortured him to his heart's content, much to the annoyance of the medical gentlemen and the amusement of the court.

It is important that we should remember, that in all contested cases of lunacy, relating to the administration of property, it is a matter of moment for counsel, supporting the commission, if he cannot exact an admission of insanity, to induce the witness to acknowledge the existence of an incapacity (apart from the presence of actual lunacy) to manage both the person and property. If the question is: "Do you consider the party of unsound mind?" and the answer should be either negatively, affirmatively, or of a doubtful character, the witness, in all probability, will be immediately asked, "Do you consider the party capable of taking care of himself, and of managing his property?" Upon one occasion, a question of this character was put to myself. "Yes, *legally* competent." "Legally competent!" echoed Sir F. Thesiger; "pray, sir, leave *us* (the lawyers, of course) to decide that point." He was most anxious to force from me an admission, that, in the ordinary acceptation of the term, the party was not in a condition to take care of herself, or to manage her property; but drawing what I conceived to be a psychological distinction between *natural* and *healthy incapacity*, and *the incapacity the effect of insanity*, I refused to make the admission he was anxious to obtain, and which, if procured, would, I have no doubt, have been turned adroitly against me. It was upon the same occasion, and during the same inquiry, that I was asked, whether, if *I* thought the party were competent to manage herself and her affairs, the *world* would be of the same opinion? I replied, "that, upon intricate and disputed questions of science, I did not think the opinion of 'the world' a safe guide." Upon which Sir F.

Thesiger rejoined, "*Then, I presume, you look down upon the opinions of the world?*"* If I had been permitted, I might have quoted in justification of my remark, the sentiments of a modern philosopher of no mean repute: "The general voice of mankind, which may often serve as a guide, because it rarely errs widely or permanently in its estimate of those who are prominent in public life, *is of little value when it speaks of things belonging to the region of exact science.*"† The opinion of the majority upon questions within the comprehension and grasp of men of ordinary intelligence and natural sagacity, is entitled to our profound respect. It may be, and often is right. But does not history satisfactorily establish, that what in common parlance is designated as the "generally-received opinion" is occasionally very remote from the truth?

"Interdum vulgus rectum videt, est ubi peccat."—Hor.

There is a *legal* incapacity, and, according to law, it is the consequence of diseased, or unsound mind. There is also ordinary and natural incapacity, which may co-exist with a healthy and a sound understanding. This important and essential distinction, the medical witness should never overlook, when giving his evidence.

Having offered some advice to the witness relative to his general deportment whilst recording his evidence, and endeavoured to convey to him some conception of the legal and psychological import of the term "unsoundness of mind," I would take this opportunity of making some remarks upon the importance of avoiding a vague and indefinite application of this phrase. We should enter the court with a clear, precise, and scientific appreciation of the *medical import* of the term. This is most essential to our credit. An indiscriminate and lax use of the word is *invariably* used to our disadvantage and discomfiture. I have seen the most able medical witnesses break down, in consequence of neglecting to be cautious in this particular.

It was at the commission of lunacy instituted with the view of

* I should regret if any of my readers for one moment imagined that I in the slightest degree complain of the course of examination pursued by this able, honourable, and justly distinguished advocate. The conduct of Sir F. Thesiger during the painful and protracted inquiry into the sanity of Mrs. Cumming, is beyond all praise. In his zeal for the interests of his client, he never deviated from the deportment of the gentleman.

† History of the Inductive Sciences, by Dr. Whewell.

annulling Miss Bagster's marriage with Mr. Newton, on the ground of imbecility, that Dr. Haslam made his celebrated declaration as to his belief in the universality of unsoundness of mind.* Whilst being examined by the present Lord Chief Baron, then Sir F. Pollock, Dr. Haslam was asked the following questions :—Q. Is she (Miss Bagster) of sound mind? A. I never saw any human being who was of sound mind.—Q. That is no answer to my question. A. I presume the Deity is of sound mind, and He alone.—Q. Is that your answer? A. I presume the Deity alone of sound mind.—Q. How many years have you been a mad-doctor? A. About forty.—Q. When did you learn that the Deity was of sound mind? A. From my own reflections during the last fourteen years, and from repeated conversations with the best divines in the country.—Q. Is Miss Bagster of sound mind? A. Competently sound.—Q. Is she capable of managing herself and her affairs? A. I do not know what affairs she has to manage.—Q. How often have you given evidence before commissions of lunacy and before a jury? A. I cannot tell. I don't know.—Q. Have you any notion? A. Notion is very much like knowledge.—Q. Have you any idea? A. An idea is a visible perception and a direct recollection.—Q. Have you any belief? A. I cannot say that I have any belief, for that is a direct recollection.†

To say nothing of the impropriety and bad taste of the witness involving himself in a contest about words, and thus fencing with counsel, I would observe, that had Dr. Haslam recognised the prin-

* Sir W. Follett observed, when commenting upon this declaration, "that Dr. Haslam had only followed in the wake of Lord Ellenborough, who, during the trial of Mr. Perry, of the *Morning Chronicle*, for a libel in ascribing mental imbecility to the late King George III., remarked that it was no libel to ascribe to any man unsoundness of mind, *for none, save the Deity, was of perfectly sound mind*."

† During a debate in 1843, in the House of Lords, on the subject of "*Insanity and Crime*," Lord Campbell, in course of his speech, said, "I know a very distinguished medical practitioner, Dr. Haslam, who maintained, not that there were many who *were* more or less insane, or that all of us *had been* insane at one period of our lives, but that *we all were actually insane*."

*Lord Brougham*.—"I have heard him say it."

*Lord Campbell*.—"I, too, have heard him say it repeatedly, and Dr. Haslam would have been ready to prove it."—*Hansard's Parliamentary Debates* for 1843, vol. lxvii. p. 741.

Need we, after such a declaration, feel any surprise at the attempts made to repudiate medical testimony in cases of insanity?

ciple to which I have given exposition, and, in reply to the interrogatories, refused to allow the existence of any unsoundness of mind that was not the *direct result or offspring of disease*, an unfortunate admission, like that to which I have referred, and with which medical witnesses, in cases of insanity, have so often been twitted, never would have been made. If this physician had qualified his opinion by stating that, according to his observation and judgment, there were few minds in a perfect state of development, well-balanced, and disciplined, without some natural eccentricity, or weakness, or in which some one or two ideas had not obtained a predominance, and exercised an influence incommensurate with their value, he would only have given expression to sentiments in conformity with the general experience of all thinking men; but having been appealed to by the court, as an *expert*, and a man of science, to decide the solemn questions of sanity and moral responsibility, it was imperative upon him to have been more guarded and precise in the use of terms having a recognised, popular, legal, and medical import. Dr. Haslam's absurd dogma may be in harmony with the "melancholy madness of poetry,"* and in unison with the fanciful creations of the novelist, but it is certainly not in accordance with the calm speculations of the philosopher.

"'All men are mad,' the raging poet cries;
Each frantic reader, 'not quite all,' replies;
Lifting his jaundiced eye, 'not all, sir, sure,'
Cries rich Avaro, 'mad beyond all cure;'
'Not all,' coy Chloe adds, by wine made bolder;
'Not all,' repeats the parrot, from her shoulder;
The pensioned peer affirms, 'it is not so;'
The mitred politician echoes, 'no!'
Each for himself and friends, the charge denies,
And Bedlam joins to curse poetic lies."

"Disorders of the intellect," says Dr. Johnson, "happen much more often than superficial observers will easily believe. Perhaps, if we speak with rigorous exactness, no human mind is in its right state. There is no man whose imagination does not sometimes predominate over his reason, who can regulate his attention wholly by his will, and whose ideas will come and go at his command. No man will be found in whose mind airy notions do not sometimes tyrannize, and force him to hope or fear beyond the limits of sober probability. All power of fancy over reason is

* Junius.

a degree of insanity; but whilst the power is such as we can control and repress, it is not visible to others, nor considered as any deprivation of the mental faculties; it is not pronounced madness, but when it becomes ungovernable, and apparently influences speech and action."*

In this passage the celebrated moralist uses the terms "insanity" and "madness" in their popular and vulgar signification, irrespectively of any attempt at psychological accuracy, or exactness. But the medical witness is not, in the slightest degree, justified in adopting the dicta of Dr. Johnson, or any other writer, however elevated his status in literature, science, and philosophy, who thus unscientifically, vaguely, and indiscriminately uses these important medico-legal terms. But medical men are not alone censurable for attaching to this phrase a general and an unphilosophical acceptation. Eminent legal writers—distinguished members of the bar—celebrated statesmen—following the example of the great lexicographer, have talked of insanity and unsoundness of mind without any regard to the right acceptation of the words. In the eloquent speech of the Solicitor-General during the trial of the Earl Ferrers for the murder of his steward, the following observations occur :—" Every violation of duty proceeds from insanity. All cruelty, all brutality, all revenge, all injustice, is insanity; there were philosopers in ancient times who held this opinion as a strict maxim of their sect, and I consider the opinion right in philosophy, but dangerous in judicature. It may have a useful and a noble influence in regulating the conduct of men, in inducing them to control their impotent passions—in teaching them that virtue is the perfection of reason, or reason is itself the perfection of human nature—but not to extenuate crimes, nor to excuse those punishments which the law adjudges to be their due." Here again we perceive the error into which the most distinguished men in the legal as well as in our own profession have fallen, by refusing to recognise the great psychological fact, that *no mind can properly be considered to be " unsound" or " insane" which is not subject to actual disease,* the " insanity" and " unsoundness" being invariably the products —the effects—the consequences, of some deviation from the healthy condition of the brain, its vessels or investments, disordering the mental manifestations.

* Rasselas.

Having previously explained what I conceive to be a right definition of the term delusion,—if a definition of the word be practicable, and within the genius of our language,—and having, I hope, clearly and conclusively established, that the non-existence of a delusion is no proof of the *absence* of insanity, unsoundness of mind, and legal irresponsibility, I would, with submission to those who may be called upon in our courts of justice to give evidence in these important cases, offer a few suggestions respecting the legitimate medical interpretation of this disputed phrase. Much of the conflicting character—much of the discredit which has, alas! attached to medico-legal evidence—much of the odium and obloquy thrown upon the examinations of medical men in disputed cases of insanity—may, undoubtedly, be traced to a want of a right and philosophical appreciation of the terms we employ whilst recording our testimony. The word delusion has been exposed to much abuse. No two witnesses appear to have the same conception of the phrase, and consequently advantage is taken of this discrepancy of opinion, and evidence which ought to be considered as extremely valuable, has, in reality, little weight with the court.*

The word delusion is often improperly used to express an erroneous conception, a wrong deduction, an illogical conclusion, a false inference, a palpable fallacy, an unphilosophical result. It is unnecessary for me to remark, that no mind, however well-organized, whatever may have been its degree of training, or the extent of its knowledge, is free from such healthy and normal aberrations. The philosophical opinions of one era are succeeded by those of the following epoch; one sect of philosophers triumphantly overturning the brilliant theories and speculations of those that preceded it. Fashion, peculiarity of education, caprice, social, moral, and political conditions, all may greatly influence, and often do operate, not only in modifying the pre-

* Much has been said of the want of unanimity of opinion among medical men of admitted science and experience in reference to questions of insanity. Is it possible, or even desirable, to have uniformity of sentiment? "I have heard," says Lord Campbell, in his "Life of the Earl of Eldon," "his lordship cite with great glee a saying of Lord Thurlow, that the decrees of the Scotch judges were least to be respected when they were unanimous, as in that case they, probably without thought, had followed the first of their number who had expressed an opinion, whereas, when they were divided, they might be expected to have paid some attention to the subject."

vailing opinions and ideas of individuals, but of large sections of society, as well as of nations themselves; thus inducing trains of thought, and mental sequences, apparently inconsistent with our modern ideas of healthy regularity or even sanity of mind. The superstitious notions and practices of the Brahmins, and of the inhabitants of many portions of the uncivilized world, may appear to us to indicate insanity and unsoundness of mind. But are we justified in this opinion? The general belief, once entertained, of the possibility of curing, by means of the royal touch, a most loathsome disease; the credence attached to the trial by "ordeal of touch," and to witchcraft, even by men of great intellect and learning, holding the highest judicial positions in the country,—were compatible with healthy and rational understandings. Even in our own time, men, whose sanity of mind cannot for a moment be questioned, arrive, by what *they* conceive to be a cautious and philosophical process of induction, at the most absurd conclusions, paradoxes, and fallacies, in open violation of all the elementary rules of logic, right principles of ratiocination, and obviously at variance with the views generally entertained by truly philosophic, thinking, and reflecting men. But are we justified in designating these false inferences, defective reasoning, illogical conclusions, arrogance, conceit, and folly, as *delusive*, and *therefore* as indicative of insanity? A man, in a healthy state of mind, may believe himself capable, in certain exalted conditions of the nerves of sense, of seeing through the *epigastric* region, or a nine-inch brick-wall! He may also consider it possible under the influence of the phenomena of mesmerism, to transfer his spirit into another state of existence,—and, after placing the party to be operated upon under mesmeric influence, to substitute his own volition for the will of another. If I were asked in a court of justice whether I considered chimeras and monstrosities like these to be delusions, I should unhesitatingly reply, *that they were not so, in the right acceptation of the term.* In common parlance they are vulgarly so denominated, but speaking, as we ought always to speak when in the witness-box, with a proper appreciation of the science of psychology, and the philosophic and philological import of terms, I would suggest, that *no notion of the mind, however ridiculous, illogical, fallacious, and absurd, should be admitted to be a delusion, or evidence of unsound mind, unless it be obviously*

*and unmistakably the product of a diseased intellect.* It is the object of counsel to confound the medical witness; to obtain from him an admission that certain extravagant opinions and anomalous articles of belief are delusions and symptoms of insanity; and selecting, perhaps, the most unphilosophical results at which men have arrived, the witness is requested to say, whether, in his estimation, they are not morbid exaggerations of the fancy, delusions, and evidences of mental derangement? A physician was asked, during a judicial inquiry as to the sanity of a party, whether he believed in the so-called phenomena of mesmerism? He replied in the negative. He was then interrogated whether he did not consider a man to be under a delusion who could bring his mind to believe that, whilst in a mesmeric trance, he could see through a nine-inch brick-wall? The physician immediately answered, that such would be his impression. Having obtained this unfortunate admission, the counsel proceeded to prosecute his examination, and the following questions were then put:—Q. Are you not aware of the existence of a section of educated and scientific men who firmly believe in the truth of mesmeric phenomena? A. Yes.—Q. Do they not consider it possible to see without the aid of ordinary vision? A. Yes.—Q. Are there not a few medical men of repute who have given in their adherence to this opinion? A. Yes.—Q. Do you know Dr. ——? (mentioning the name of a physician of great repute). A. Yes.—Q. Are you not aware that *he* has publicly professed his belief in the existence of what you term a delusion? A. Yes. —Q. Then it is your opinion that Dr. —— is of unsound mind? The witness at once perceived the dilemma in which he was placed, by not recognising the distinction between a false conclusion, an illogical and unphilosophical deduction, and those conceptions or delusions of the *diseased mind,* the products of insanity, and was unable to escape from the grasp of the acute lawyer, without materially damaging his evidence. The counsel, in his address to the jury, was not forgetful of this admission, and with indignant eloquence asked, what credit they could attach to the opinion of a witness who pronounced men of established repute, in consequence of their belief in mesmerism, to be under the influence of a delusion—in fact, to be of unsound mind?

If this gentleman had entered the witness-box with a philosophic appreciation of the import of the word, no ingenuity or special pleading of counsel, however exalted his reputation for legal subtlety, his expertness in the cross-examination of witnesses, and adroitness in obscuring the truth, would have induced him to fall so readily into his power. I again advise the medical witness never to admit any idea to be delusive, unless it be obviously and palpably the offspring, the product, not of a mind unevenly balanced, with a natural disposition to distort facts, believe in bad logic, or in any gross absurdity of the day, but *of an understanding perverted by disease.* Healthy minds, sane understandings, vigorous intellects have been known to imbibe the most extravagantly false notions, and to arrive at the most outrageous results, and to be subject to the most extraordinary idiosyncrasies of thought and feeling. These must be denounced and exposed as absurd, dangerous, and unphilosophical deductions or principles of belief; but let us not pervert the use of language by designating them as *delusions,* and adduce them as proof of insanity! The term "healthy delusion," which has been occasionally used by men of scientific eminence, when discussing these questions, is equivalent to the phrase "*healthy* unsoundness of mind," and "*normal* insanity."*

There are other occasions requiring the evidence of the members of our profession before we are warranted in interfering with the liberty of the subject. By various Acts of Parliament enacted for the purpose of regulating the confinement of persons on the ground of insanity, it is wisely provided that no step of this nature is legal unless under the sanction of two medical certificates. The power so invested in the hands of two legally qualified practitioners has been made the subject of much comment and animadversion. It has been said, that the legislature is not justified in thus placing the freedom of the citizen at the mercy of two professional gentlemen, who may either be incompetent from ignorance to decide the question of insanity, or may be agents in the hands of unprincipled relations or designing friends, who may, from sinister motives, be desirous of depriving him of his free agency, and the control of his

* In the celebrated Commission of Lunacy upon Mr. Davies, Dr. Haslam was much laughed at for talking of the alleged lunatic having a "delusion of manner!" Lord Brougham was extremely happy in his comments upon this unfortunate expression.

property. With the view of meeting this popular objection, various modifications of the law have been suggested. It has been proposed that, previously to the actual confinement of the alleged lunatic he should be taken before a magistrate or a judge of an inferior court, and that the case should be submitted to the consideration of a jury prior to the certificates of the medical men being acted upon! Again, others who feel more strongly upon this question, and who denounce all confinement, except in cases of acute insanity, accompanied by acts of great violence, as monstrous and unjustifiable outrages, propose that, in every case, a commission of lunacy should issue, for the purpose of considering, whether the party represented to be insane be sufficiently so to justify his being placed *in duresse.* With deference to those who have originated these suggestions, I am bound to declare them to be totally impracticable. There are many cases of insanity requiring to be placed under temporary surveillance and proper medical and moral treatment which could not be exposed to any of these preliminary ordeals without imminent danger to life, or without seriously interfering with the safety of the patient, and perhaps altogether retarding his recovery. In many incipient forms of insanity, where the symptoms are acute and associated with much physical disturbance, a speedy re-establishment of health may generally be expected if the patient be removed, temporarily, from the morbid associations of home, and immediately brought within the sphere of systematic medical treatment. In cases of this description, a non-medical jury or judge, ignorant of the character of these affections, and unable to detect the nice shades of incipient insanity, or to recognise the immense importance of prompt and energetic treatment in the early stages of this disease, would, in all probability, from a sense of justice, refuse to sanction confinement of any description, unless in cases of glaring, violent, palpable, mental derangement. No judge and jury, however upright in character, and honest in intention, can be considered qualified, unassisted by medical evidence, to adjudicate in these important and delicate cases, unless they have acquired, by patient study and long-continued practical observation, an intimate knowledge of the varied phases and subtle phenomena of mental disease. When referring to the charge of an anonymous slanderer, that some medical men, from their poverty, might be bought over to sign the fatal document by the bribes of avaricious relatives, it has been justly observed that, "Although abuses have taken place, we do not believe there ever existed any ground for such an imputation as this; and we are quite satisfied that, in the present day, if no other principle restrained a man from granting a certificate improperly, the certainty of detection would deter him. If the case were to be considered by a jury or county judge, as a preliminary step to confinement, there would be no end to litigation and expense. One half of the alleged lunatic's estate would go to settle whether he should be confined, and the other half under a commission to determine whether or no he was a fit subject for interdiction!"

But let me ask, whether the power so invested in us by the statute law is abused, and whether any necessity exists for legislative interference? Judging from my own experience of documents of this character, I can truthfully affirm that I have never seen an instance—a solitary example—in which the practitioner was not fully justified in certifying, not only to the existence of insanity, but to insanity of such a kind and degree as to justify immediate surveillance. To the honour of our much-slandered profession, I would add, that I firmly believe, as a body of men constituting an important section in the community, we are scrupulously, conscientiously, cautious and exact in the exercise of this power, and that the instances of abuse are so rare, that it would be an act of great injustice to throw, by any alteration of the law, any doubt upon the honesty and integrity of our profession. I trust the day may never arrive when legal will be substituted for medical authority in these cases, and a non-professional judge or a jury be empowered to interfere with the legitimate functions of the medical practitioner! Surely we are, by education, habits of thought, knowledge, and experience, peculiarly fitted to solve the intricate and knotty point involved in the elucidation of doubtful cases of insanity. Sad will be the day for our science when the medical, moral, or judicial care of the insane is transferred from the hands of the medical profession to those of the barrister, highly as I respect his honourable vocation.

Having made these preliminary observations relative to an important part of the subject, I now proceed to refer more specifically to the duties devolving upon the profession when called upon to certify in cases of alleged mental incapacity, prior to the removal of the patient to a place of confinement. The law wisely requires the production of two medical certificates, not only of insanity, but of insanity to such an extent as to justify restraint, either in private lodgings or in public or private asylums. The Act of Parliament makes the preliminary step imperative *under all conditions of moral restraint,* on the ground of insanity, excepting when the person is confined in *his own* house, or is placed under the care of one who receives no payment for his support. No insane person can be legally controlled in a private house or lodgings without an order for his detention is filled up and signed, or without two medical certificates. The Act of Parliament also requires that *every person* receiving and taking charge of an idiot, lunatic, or party of unsound mind, should make an official return of the fact to the Commissioners in Lunacy.

Great caution is necessary before, under such circumstances, certifying to insanity. In the majority of cases in which we are called upon to testify to the existence of lunacy, the derangement of mind is generally so obvious, and is accompanied by such violence, extraordinary delusions, and excitement, that the medical man has little or no hesitation in complying with the provisions of the statute, and of immediately signing the necessary legal document. But cases do occasionally occur in which much prudence, judgment, and great caution are requisite. Statements may be made to the medical practitioner by the relatives of the alleged lunatic, which, if true, clearly indicate the necessity for prompt interference; but it is our duty to avail ourselves of every reasonable opportunity of ascertaining, not only whether certain facts have not been exaggerated, but whether there is any truth in the evidence adduced to us as proof of the presence of mental derangement. In signing a certificate of lunacy, it should never be forgotten that we may, even at a distant period, be called upon to defend the act in a court of law. This renders imperative, great caution and careful inquiry, in every case presented to our notice.

If it should be alleged that the patient has been guilty of acts of violence, ascertain under what circumstances they were committed. Also inquire whether there has been any reasonable provocation, and if he has acted under the influence of a delusion, natural violence and impetuosity of temper, or has been justified by *actual circumstances, in so committing himself.* If insane, he may be guilty of an outrage quite disproportionate to the exciting cause. Under the impression that a person supposed to be insane, was inclined recklessly to squander his property, a member of the family or friend might feel himself justified in secreting the patient's cheque-book—in placing his private papers in a position of security. A knowledge of these facts may, in a person of irritable temper, and perfectly sound condition of mind, induce great irritation and provocation, and probably lead to acts of violence and resentment; but if, influenced by such a cause, the patient were to procure a pistol or a knife, with the object of revenging himself for such an apparent insult and interference with his private property, we could not consider this, coupled with other symptoms, otherwise than suspicious evidence of insanity, justifying protection. Insanity often exhibits itself in an unhealthy exaggeration of actual circumstances, conditions, or facts. Should the person accuse others of robbing him, ascertain, as far as is consistent with the respect due to those about the patient, whether there is any foundation for the statement. In some cases, it is difficult to arrive at the truth ; but it is our bounden duty, our solemn obligation, to fully inquire into every particular likely to throw light upon the case before interfering with the liberty of a fellow-creature by certifying to his insanity. In some instances, the alleged lunatic, fully sensible of the object of the professional man's visit, and knowing what ulterior measures are to be adopted, will set the medical examiner at complete defiance, and resolutely deny all the representations of those about him.

I had to examine a remarkable case of this nature. I was requested to see a gentleman who was said to be suicidally insane. Upon inquiry, I ascertained, from good authority, that under the influence of most distressing hallucinations he had attempted to hang himself. The patient firmly, earnestly, and apparently with great truthfulness, resolutely and repeatedly denied the fact. He declared that it was an invention—a pure creation of the imagination, originating with his family; that he was happy, subject to no depression, had a strong wish to live, and great fear of death. I examined him, in conjunction with another physician, and neither of us could seize hold of the salient point, or satisfy himself that the man was actually insane. But we asked ourselves, what motives could his family have for thus misrepresenting the facts of the case? We felt quite assured, from the character of the evidence presented, that an attempt at suicide had been made; but the patient, with an ingenuity which would have reflected credit upon a *nisi prius* lawyer, parried, with great skill, all the questions, and gave such prompt and happy replies to our anxious interrogatories, that we were compelled to admit ourselves, for a time, perfectly defeated. By a course of conversation, I drew the gentleman's thoughts into a different channel; and whilst my attention was apparently directed elsewhere, I kept a close watch upon all his movements. I perceived, as I imagined, some kind of instrument projecting from his pocket. He perceived that my eyes were directed to this, and he immediately expressed an earnest wish to leave the apartment. I at once said, "I cannot permit you to do so, until I know what you have concealed in your trowsers' pocket." He at once manifested signs of embarrassment and excitement, and rising rapidly from his seat, endeavoured to rush out of the door. He was immediately prevented from doing so, and his pockets emptied, and a razor discovered. In his pocket-book a letter was found, which he had written the same day, and addressed to the coroner, intimating to him that he was pursued by an evil spirit, and this impression had driven him to commit an act of self-destruction! Fortunately for our own reputation, and for the patient's life, this providential discovery was made.

It may be necessary to see and examine the patient on more than one occasion before the physician is satisfied as to the actual state of his mind. In cases of doubtful character, I would suggest that this course should invariably be adopted, taking the necessary precaution to recommend close vigilance during the interregnum. I suggest this course, in consequence of my being acquainted with the case of a lady, whose removal from home was for a few days temporarily postponed, in compliance with the cautious and judicious advice of the medical man, who admitted that he could not detect, according to his apprehension, sufficient evidence of insanity to justify him in signing the certificate. During the interim, she succeeded in destroying herself! In a few instances we are justified in partially acting upon the representations of the family and friends of the alleged lunatic. If a delusion be detected, it must be referred to; and if the patient has committed any overt acts of violence, or manifested a suicidal disposition, it is our duty to refer to these facts, guarding ourselves by stating, that we have derived such information from parties immediately about the patient. It is important, in all cases, to specify the character of the existing delusion. The expression of a belief in the fact of delusive ideas, and of the presence of abstract insanity, without a specification of facts, renders a medical certificate invalid. I have often seen certificates worded to this effect: "I have formed my opinions from the fact of the party being insane"—"being under delusions"—"being excited"—"being violent." These generalizations should be carefully avoided: the more concise the account of the patient's condition, the closer will it be in unison with the expressed wish of the Commissioners in Lunacy. The record of one clear and unmistakable delusion is quite sufficient for all legal purposes. But cases do occur where no delusion can be detected, and yet confinement may be absolutely necessary. Under such circumstances, it is the duty of the medical man to enter more into detail as to the facts of the case. Perhaps I may be excused for suggesting, that in every instance of this kind, the parties should keep copies of their certificates.

Having, I think, conclusively established that we have no uniform legal or medical test of insanity to which we can safely appeal in criminal cases, you will ask, have I any psychological *criteria* to suggest for the safe guidance of the profession?—can I propound any principles which will assist the medico-legal witness in arriving at a satisfactory result? In reply to these interrogatories, I allow that we have no infallible standard, no certain principles which would admit of general and indiscriminate application. The only safe rule upon which we can act, is that of comparing the mind of the alleged lunatic, at the period of his suspected insanity, with its prior, natural, and healthy manifestations; *to consider the intellect in relation to itself*, and to no artificial *à priori* test. Dr. Haslam suggests that the mind of the physician should be the standard by which the sanity should be determined; but this is presuming the mind of the physician to be healthy and sound. In the language of Dr.

Combe, "the true and philosophical standard in all cases is the patient's own natural character, and not that of the physician or the philosopher. It is the prolonged departure, without an adequate external cause, from the state of feeling and modes of thinking *usual* to the individual when in health, that constitutes insanity in the true medical acceptation of the term." This portion of my subject is, however, too comprehensive in its character to admit of elucidation in this lecture.

I have endeavoured in the preceding observations to place before you a sketch—a mere outline—of the character of the evidence admissible in our civil, criminal, and ecclesiastical courts, in cases of disputed lunacy, and I have, to the best of my ability, but still I fear very imperfectly, delineated the duties—the anxious functions—specially devolving upon us, when, in the exercise of one of our responsible vocations, we are called upon for our opinion as medico-legal witnesses in cases of alleged insanity. There is, unhappily, a prevailing prejudice—an illiberal feeling—manifested towards those whose province, and, I may add, whose happiness and privilege it is to stand prominently forward, upon these occasions, to aid by their evidence the administration of justice, under circumstances peculiarly solemn and affecting. These sentiments are not restricted to persons ignorant of the great truths of psychology, and of the characteristics of deranged mind, but they are, to some extent, participated in by a few narrow-minded men among ourselves, who, from motives difficult to divine, evince a disposition to disparage the benevolent and Christian efforts of those who, in the discharge of an imperative professional duty, are ever ready to interpose between the insane criminal and the dreadful and terrible punishment of the law. It may be argued, that this feeling, both in and out of the profession, has been the result of a disposition on the part of the medico-legal psychologist to sanction by his evidence an unphilosophical, dangerous, and a lax use of this plea. If such a tendency has been exhibited, may it not have been the effect of the most benevolent motives—the offspring of truly noble aspirations?—have originated in feelings that do honour to human nature?—have arisen from a conviction that it is our duty to temper justice with mercy, and from a strong conviction that, in obedience to one of the great principles of British Jurisprudence, we are bound, upon all occasions, to give to the unhappy culprit the benefit of any doubt that may arise respecting his sanity and legal responsibility? In considering this question, we should never forget in many criminal cases the alliance to insanity is close—the line of demarcation between the two conditions indistinct, vague, and shadowy—the boundary separating crime from insanity obscure—the one state often, almost imperceptibly, blending with the other, and that the facts associated with the criminal act so analogous to the recognised phenomena of mental disease, that the medical witness, feeling that in his hands is deposited the life of a fellow-creature—that upon his evidence depends the decision, whether the extreme penalty of the law is to be carried into effect—he, under the conflicting and painful emotions which such a position is calculated to call into active exercise, hesitates in consigning a fellow-creature to an ignominious death, if he can, without doing violence to his judgment and conscience, record his opinion in favour of the prisoner's insanity.

We have only to glance the eye over the tabular statement suspended near me, in order to form a correct idea of the relationship between the criminal and the insane mind.* The table to which I now refer was not drawn up designedly to establish this position; but does it not clearly prove—forcibly establish—the painful fact, that there is in existence a large amount of crime closely connected by hereditary predisposition and descent with diseased mind? Does not a recognition of this truth establish to us, as Christian philosophers, the necessity of cultivating more benevolent feelings, a more enlarged and expansive philanthropy, towards those who, if not morbidly impelled to the commission of crime by an originally malformed cerebral organization, inherit from their parents a marked predisposition to irregularity of thought and action, which ought to appeal—powerfully appeal—to us when estimating the degree of moral guilt attached to any deviation from our *à priori* notions of healthy intellect, or strict moral rectitude? I maintain, and facts—an overwhelming mass of facts—clearly, irresistibly, and conclusively demonstrate my position,—that there is a vast amount of crime committed by persons, who, if

* Vide Table, at the end of the lecture, showing, in numerous cases, the close alliance between crime and insanity.

not "legally" or "medically" insane, occupy a kind of *neutral ground between positive derangement and mental sanity.* I do not broach this idea with the view of supporting the absurd, unphilosophical, and dangerous opinion, that *all* crime is more or less referable to aberration of mind; but I do affirm, that in estimating the AMOUNT OF PUNISHMENT to be awarded, it is the solemn duty of the judge, not only to look at the *act itself,* but to consider the *physical* condition of the culprit—his education—moral advantages—prior social position—his early training—the temptations to which he has been exposed—and above all, WHETHER HE HAS NOT SPRUNG FROM INTEMPERATE, INSANE, IDIOTIC, AND CRIMINAL PARENTS.

"The little I have seen of the world," says an able writer, with a capacious heart, overflowing with love for his fellow-creatures—"the little I have seen of the world and know of the history of mankind teaches me to look upon the errors of others in sorrow, not in anger. When I take the history of one poor heart that has sinned and suffered, and represent to myself the struggles and temptations it has passed—the brief pulsations of joy—the feverish inquietude of hope and fear—the tears of regret—the feebleness of purpose—the pressure of want—the desertion of friends—the scorn of the world, that has little charity—the desolation of the soul's sanctuary, and threatening voices from within—health gone—happiness gone—even hope, that stays longest with us, gone,—I have little heart for aught else than thankfulness that it is not so with me, and would fain leave the erring soul of my fellow-man with Him from whose hands it came."*

In venturing, with great submission, to make these observations, after offering my grateful thanks to the President, Council, and Fellows of this learned Society, for the courtesy, kindness, and generous indulgence which have been manifested towards me during my period of office, I would, in conclusion, protect myself from the imputation of giving utterance to—of breathing the faintest semblance of—an expression that would justify a doubt as to the existence in my mind, of a feeling of deep reverence, and profound respect, for those great and illustrious men, whose unrivalled erudition—brilliant attainments—fervid,

* Hyperion, by Longfellow.

glowing, and impassioned eloquence—world-wide reputation—whose universally acknowledged public and private worth, must, as long as the mind retains its appreciation of virtue, its love of liberty, and admiration of genius, be closely identified, and indissolubly associated, with the brightest and most hallowed periods of the constitutional, parliamentary, and legal history of our country. But may I not ask, whether, since the times of Lord Coke, Sir Matthew Hale, Judge Blackstone, Lord Hardwick, Lord Mansfield, and Lord Chancellor Erskine, we have made no progress in the important truths of medical-psychology—have obtained no clearer insight into the phenomena of the human mind—are not more intimately acquainted with its diseases—and do not entertain more benevolent, just, philosophical, and enlightened views of the great subject of crime, and of the principles of civil and constitutional law?

Can we set bounds — prescribe limits — easily appreciable, well-defined limits — to the progress of knowledge? Have we not, within the last half century, made giant and colossal strides in all departments of art, philosophy, and science? Does not the genius of man indignantly repudiate all attempts to fetter its onward advance, and tie it down to the crude, exploded, and obsolete dogmas of past ages? If such be the fact in relation to the mathematical and physical sciences—to chemistry, medicine, physiology, mechanics, political and social economy, why, I ask, should the great subject now under consideration be the only exception to the general law regulating human progression? Whilst referring to the great intellects and master-minds of former epochs, as well as to the illustrious men of a more recent period, may I not exclaim,—

> "Great men were living before Agamemnon,
> And since, exceeding valorous and brave?"

... ... ... ...

### CRIME AND INSANITY.*

(*A Tabular Statement referred to in page* 129.)

| Initials of Criminal | Verbatim Extracts from Letter of Referee. | Observations on Degree of Intellect, &c., by the Chaplain when first seen. | Schoolmaster's Report on leaving the Prison. | State on leaving the Prison, as noted by Chaplain. |
|---|---|---|---|---|
| J. C. | Mother touched with symptoms of insanity. | ... ... | Improved in reading and writing. | Improved generally. |
| R. L. | Grandmother insane ... | Read imperfectly. | Read well; write imperfectly; 4 rules of arithmetic. | |
| J. H. | Sister rather weak in mind. | Only knew the alphabet | Read and write well; Rule of Three. | Very cheerful; improved in general knowledge. |
| H. N. | He and most of his family evinced symptoms of insanity. | Of the lowest kind. | Read very imperfectly; write a little; learned a little arithmetic. | Sent away incorrigible. |
| J. C. | Two sisters insane ... | Of the lowest intellect: did not know A, B, C. | Read *well;* write tolerably; 4 rules. | Somewhat improved in general. |
| D. M. | His mother subject to nervous fits. | ... ... | Read and write well; Rule of Three. | Mentally, not morally, improved. |
| J. D. | One of his family (his mother, as I have every reason to believe), labouring with insanity. | ... ... | Read and write well; 4 rules. | Improved, in religious knowledge; very cheerful. |
| R. | Of a simple turn of mind. Uncle in an asylum. | ... ... | Improved considerably ... | In Scriptural knowledge also. |
| W. J., alias W. C. B. | Skull fractured three years ago. | ... ... | Improved in reading and writing; Rule of Three. | Improved in Scriptural knowledge. |
| W. G. | Sister considered rather silly. | Of lowest intellect: did not know the alphabet. | Read and write imperfectly; 4 rules. | Cheerful. |
| A. H. L. | Had become *dejected* and *absent* after failure in business, and showed symptoms of *insanity.* | Very low in spirits. | Read and write well; Rule of Three. | Much improved in spirits; found comfort in religion. |
| J. N. | Considered rather *as an idiot.* | Very low degree of intellect. | Read and write well; Rule of Three. | Improved in general knowledge. |
| W. N. | Almost *irresponsible* ... | Of very weak intellect | Well educated previously ... | Rather improved mentally. |
| A. A. | Weakness of mind: made sport of by fellow-servants. | Low in spirits and in intellect. | Read and write well; Rule of Three. | Mentally improved. |
| F.W.K., alias A. K. | Uncle died in an asylum: another committed suicide. Father and sisters considered weak. | Low in spirits; over-active mind; disliked his trade. | Very well educated ... ... | Morally improved. |
| J. M. F. | Mother's brother is reported to be imbecile; harmless if let alone. | Of a low degree of intellect. | Read and write well; Rule of Three. | Improved in general; was recommended to be master tailor on board ship. |
| R. B., alias E. E. S., *a Jew.* | Not considered quite correct in his mind. Aunt mad for a long time. | Peculiar turn of mind. | ... ... ... ... | Greatly improved, especially in Scriptural knowledge. |
| D. M. | Considered a simpleton | Low intellect ... | Read well; write imperfectly; 4 rules. | Improved generally. |
| J. M., alias J. T. | Uncle killed himself in a fit of insanity. | Low in spirits and intellect. | Read well; write tolerably; Rule of Three. | Much improved. |
| C. J. C. | Eldest brother exhibited symptoms of insanity. | Good intellect | Well educated ... ... ... | Improved generally. |
| T. N. | Whole family eccentric; and very weak in intellect. | Weak intellect | Read and write well; Rule of Three. | Improved generally. |
| R. R. | Uncle's intellect affected at times. | Low intellect; only knew the alphabet. | Read well; write imperfectly; 4 rules. | Improved generally. |

* From No. 163 of the "Quarterly Review."

| Initials of Criminal | Verbatim Extracts from Letter of Referee. | Observations on Degree of Intellect, &c., by the Chaplain when first seen. | Schoolmaster's Report on leaving the Prison. | State on leaving the Prison, as noted by Chaplain. |
|---|---|---|---|---|
| J. T. | Father died a lunatic ... | Ordinary intellect. | Reads and writes well; Rule of Three. | Very much improved in general. |
| J. S. | I have thought, and more, I am sure, that at times he was not altogether right in his head. | More than ordinarily reserved and very dull. | Read tolerably; wrote imperfectly; improvement very little. | On the whole rather improved. |
| H. C., alias L. | The prisoner's conduct, more especially his wandering propensities, are irreconcilable with perfect sanity. | A good intellect; apparently much compunction for sin. | Could read and write well; considerably advanced in the higher rules of arithmetic. Improvement tolerably fair. | Improved very much. Found peace and comfort in the Gospel. |
| G. R. | He was not quite sound in mind, and sometimes not conscious of what he was about. His own sister destroyed herself. | A very low-spirited man. | Could read and write very well; considerably advanced in the higher rules of arithmetic; intelligent. Made fair improvement. | Improved in spirits. Found comfort in religion also, I think. |
| W. H. | His mother has evinced symptoms of insanity within the last three years. | Nothing at all peculiar. | Read well; wrote tolerably; higher rules of arithmetic. Improvement tolerable. | Improved very much, especially in the memory. Gave himself to learning hymns, chapters, &c. |
| H. L. | His father was subject to fits. | Very low spirited | Could read and write well; mensuration. Improvement tolerable. | Very down-hearted; would have sunk here, I think, but for some religious hope. |
| J. B. | One member of the family has exhibited symptoms of insanity. | Ordinary... ... | Read well; wrote tolerably; knew the common rules of arithmetic. Very much improved. | Improved. |
| H. B. | I have known the prisoner to have fits when over-fatigued. | Ordinary... ... | Read well; wrote tolerably; common rules of arithmetic. Improvement tolerable. | Very cheerful. |
| J. K. | He received an injury in his head, from which time he became flighty and unsteady. His father was in some measure imbecile in both body and mind. | A very active mind, but most perverse. | Could read and write well; higher rules of arithmetic. Improvement tolerable. | Cultivated his mind assiduously, but was very perverse to the last. |
| W. S., alias R. | Has found him a little insane at times; he was kicked by a horse in the head. | Ordinary... ... | Could read well; write tolerably; knew the first 4 rules in arithmetic. Improvement little. | Rather improved. |
| W. F. | I knew him to labour under a severe nervous fever for several months, which I always observed afterwards to cause a lowness of spirits. It was about 8 years since. | Good, but his constitution apparently weakened by intemperance. | Read and write well; advanced in higher rules of arithmetic. Tolerably improved. | Very cheerful; much improved, I think, in every way. Gave great attention to religion. |
| J. A., alias E. W. | Has not his senses perfect. | Half-witted ... | Could read well. Made scarcely any improvement. | Rather worse. |
| W. D., alias J. B. | I fully believe him to be at times insane. His maternal grandfather died insane. | Clever; good, but perverted and abused. | Was well educated on admission. Was excused from school; improved himself tolerably by reading and private study. | Not improved. |
| W. B. | Very *soft* in many things | Low intellect ... | Could scarcely read any. Very little improved. | Rather worse. |
| J. D. | His grandmother is in a lunatic asylum. | Ordinary, but very dull. | Read well; write tolerably; first 4 rules of arithmetic. Improved a little. | Improved rather in spirits. |

